# The genus *Paris*: a fascinating resource for medicinal and botanical studies

**DOI:** 10.1093/hr/uhae327

**Published:** 2024-11-21

**Authors:** Xiao Ye, Yang Tao, Xiu-Lan Pu, Hong Hu, Jing Chen, Chun-Lin Tan, Xin Tan, Sheng-Hong Li, Yan Liu

**Affiliations:** State Key Laboratory of Southwestern Chinese Medicine Resources, Innovative Institute of Chinese Medicine and Pharmacy, Chengdu University of Traditional Chinese Medicine, Chengdu 611137, China; Industrial Crop Research Institute, Sichuan Academy of Agricultural Sciences, Chengdu 610300, China; Chengdu Medical College, Chengdu 610500, China; State Key Laboratory of Southwestern Chinese Medicine Resources, Innovative Institute of Chinese Medicine and Pharmacy, Chengdu University of Traditional Chinese Medicine, Chengdu 611137, China; State Key Laboratory of Southwestern Chinese Medicine Resources, Innovative Institute of Chinese Medicine and Pharmacy, Chengdu University of Traditional Chinese Medicine, Chengdu 611137, China; State Key Laboratory of Southwestern Chinese Medicine Resources, Innovative Institute of Chinese Medicine and Pharmacy, Chengdu University of Traditional Chinese Medicine, Chengdu 611137, China; State Key Laboratory of Southwestern Chinese Medicine Resources, Innovative Institute of Chinese Medicine and Pharmacy, Chengdu University of Traditional Chinese Medicine, Chengdu 611137, China; State Key Laboratory of Southwestern Chinese Medicine Resources, Innovative Institute of Chinese Medicine and Pharmacy, Chengdu University of Traditional Chinese Medicine, Chengdu 611137, China; State Key Laboratory of Southwestern Chinese Medicine Resources, Innovative Institute of Chinese Medicine and Pharmacy, Chengdu University of Traditional Chinese Medicine, Chengdu 611137, China; State Key Laboratory of Southwestern Chinese Medicine Resources, Innovative Institute of Chinese Medicine and Pharmacy, Chengdu University of Traditional Chinese Medicine, Chengdu 611137, China; State Key Laboratory of Phytochemistry and Plant Resources in West China, Kunming Institute of Botany, Chinese Academy of Sciences, Kunming 650201, China; State Key Laboratory of Southwestern Chinese Medicine Resources, Innovative Institute of Chinese Medicine and Pharmacy, Chengdu University of Traditional Chinese Medicine, Chengdu 611137, China; State Key Laboratory of Phytochemistry and Plant Resources in West China, Kunming Institute of Botany, Chinese Academy of Sciences, Kunming 650201, China

## Abstract

The genus *Paris*, comprising a series of distinctive medicinal plants, has been utilized globally for its therapeutic properties over centuries. Modern pharmacological studies have demonstrated that secondary metabolites from *Paris* species exhibit significant pharmacological activities, including anticancer, hemostatic, anti-inflammatory, antimicrobial, and other effects. Additionally, the unique morphological traits and large genome size of *Paris* species have continuously captured the interest of botanists and horticulturalists. Nonetheless, the conservation of wild *Paris* populations is threatened due to the lengthy reproductive cycle and overexploitation, posing considerable challenges to their development and sustainable use. This review provides a comprehensive overview of the botanical characteristics, historical medicinal uses, pharmacological effects, and toxicity evaluation of secondary metabolites in *Paris* species. It also covers the molecular biological research conducted on the genus *Paris* and proposes key research questions and important directions for future solutions. We advocate the expansion and implementation of multi-omics approaches, as well as molecular and genetic technologies recently advanced in model plant research, to intensively study *Paris* species. This will facilitate the comprehensive understanding of gene function and molecular mechanisms underlying specialized metabolite formation in *Paris*.

## Introduction

*Paris*, a genus within the Melanthiaceae family, comprises perennial herbaceous plants and is predominantly found on the Eurasian continent. *Paris* species attract considerable interest in the fields of botany and horticulture, not only due to their distinct morphological features, such as a solitary flower and a whorl of numerous leaves, but also owing to the wide variety and magnitude of their genomes among flowering plants (1C = 29.38–152.23 pg) [1, 2]. Additionally, these species are notable for their richness in steroidal saponins, which have a long history of medicinal use attributed to their diverse pharmacological properties. The medicinal use of *Paris* species was first documented in *Shennong*’*s Classic of Materia Medica* (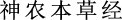). In traditional Chinese medicine (TCM), the rhizomes of certain *Paris* species, designated as ‘Rhizoma Paridis’ (RP) and listed in multiple editions of the Chinese Pharmacopoeia, are considered effective in clearing heat, removing toxins, dispersing swelling, relieving pain, cooling the liver, and settling convulsion [3]. TCM practitioners attribute various properties to it, including vermifuge capabilities and the treatment of venomous bites and infections. Clinically, RP is frequently utilized for addressing inflammatory conditions such as vaginitis, colitis, and perianal abscesses. Additionally, it is effective in treating skin disorders like flat warts, herpes zoster, and erythema, as well as in managing persistent lochia postpartum, where it demonstrates significant efficacy [4]. Moreover, RP is a primary ingredient in several marketed TCM products, including *Yunnan Baiyao* aerosol, *Gongxuening* capsules, *Jidesheng sheyao* tablets and *Yinbing xiaocuo* tincture.

*Paris* species occupy a significant position in Asia’s traditional medicine industry, largely due to their significant economic impact. In China, the cultivation of *Paris* species in Yunnan province exceeds 10 000 ha [5]. The price of RP reached 1200 CNY/kg in 2018, surpassing most other TCM materials (based on data from http://www.zyctd.com, last access date 1 October 2024). The annual sales volume of RP is ~500–1050 tons [6] and the market value of related pharmaceutical products exceeds 10 billion CNY annually [7]. *Paris polyphylla*, commonly known as 'Satuwa' or 'Satwa' in Nepal and India, has been prioritized for development of agricultural technology by the Nepalese government's department of plant resources [8]. In India, RP enjoys the reputation of being a ‘jack of all trades’, with large-scale smuggling to China and other southeast Asian countries along the India–Myanmar border [9]. Additionally, it is referred to as 'Jyuro' or 'Sokyu' in Japanese, 'Jungru' or 'Johyu' in Korean, 'Haimavati' or 'Swet Vachh' in Sanskrit, 'Tangma' in the Kham language, 'Dhumbi Mendo' by the Sherpa people, and 'A-du-ba-du' by the Monpa [8, 10, 11]. Across Asia, various parts of *Paris* species, including their rhizomes, are used in forms such as infusions, liquids, powders, and pastes for treating ailments like wounds, venomous bites, scabies, rashes, itching, and various other conditions [9, 12–14]. However, the lengthy breeding cycle of *Paris* species results in a supply shortfall that cannot meet the medicinal demand. Concurrently, long-term predatory harvesting has led to severe depletion of its wild resources, posing challenges to the conservation and sustainable use of this species.

*Paris* represents a genus of both botanical significance and medicinal value, drawing attention in the realm of plant science. With advancements in molecular biology techniques in recent years, the comprehension of *Paris* has been considerably deepened. This review encompasses current research on the botanical characteristics, medicinal value, secondary metabolites, pharmacological activities, toxicity evaluation, and molecular biology of *Paris*. Finally, we will discuss the future prospects for advancing studies on this genus.

## Botanical characteristics of *Paris* species

### Gross morphology

The genus name *Paris* is derived from the Latin *par*, meaning 'equal' or 'suitable', which refers to the distinctive two-tiered arrangement of leaves, in species like *P. quadrifolia*, where a whorl of leaves is parallel to a whorl of sepals. In China, *Paris* species are commonly known as 'Qi-Ye-Yi-Zhi-Hua’ (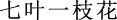, seven leaves, one flower), reflecting the typical morphology of having multiple leaves and a solitary flower. *Paris* species exhibit diverse heights varying from 10 cm to over 2 m, and have lifespans ranging from 10 to 20 years. They have thick or slender rhizomes with nodes, buds, and adventitious roots. The stem is erect, topped with a whorl of 4–20 leaves, either petiolate or sessile. The leaves are typically green and sometimes feature purple markings, with entire margins.

**Figure 1:**
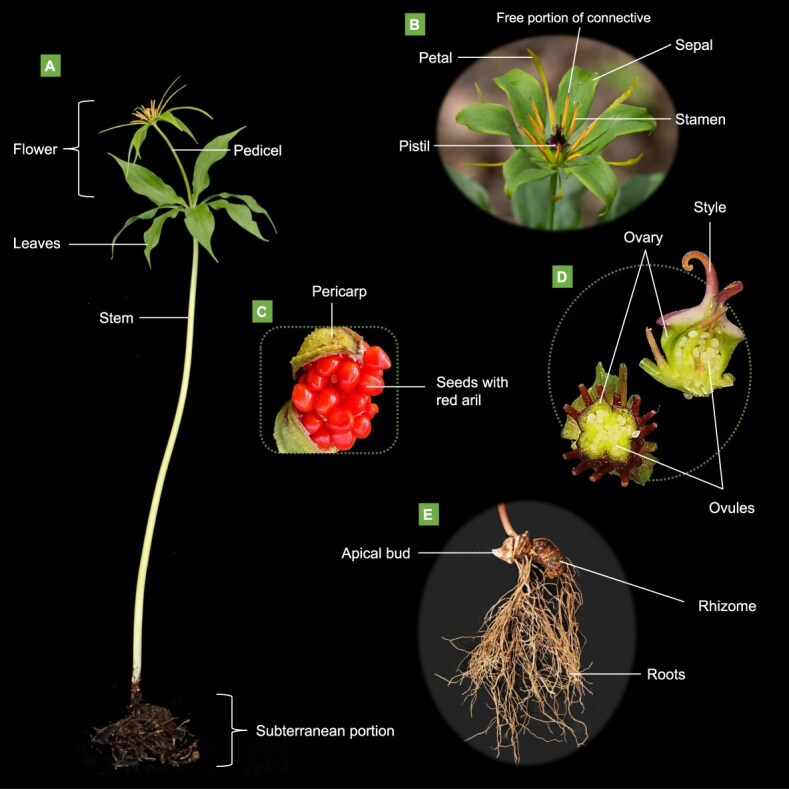
The morphology of mature *P. polyphylla*. **A** Whole plant of *P. polyphylla* in pollinating period. **B** Flower of *P. polyphylla* with typical spoon-shaped petals. **C** The mature fruit of *P. polyphylla* dehisces to reveal seeds with red aril. **D** Longitudinal section and cross-section of the ovary of *P. polyphylla* display parietal placentation. **E** The subterranean portion of *P. polyphylla* consists of a thick rhizome, slender roots, and an apical bud.

A solitary flower emerges from the whorl of leaves. The perianth is bi-whorled: the outer whorl consists of leaf-like sepals that are usually green, while the inner whorl is made up of linear or filiform petals, often yellow-green in color. The number of stamens is usually 2–4 times that of petals, with bilocular anthers and yellow pollen, and the free portion of the connective is either protruding or not, appearing linear, globular, or horseshoe-shaped, or having other forms [15, 16]. The ovary is spherical, with one locule forming a parietal placenta or multiple locules forming an axial placenta. The fruit of *Paris* species is typically ridged, a berry or berry-like capsule; unilocular fruits dehisce irregularly between placentas, while multilocular fruits do not dehisce [15]. The seeds, which are numerous, are usually covered with an aril. The typical morphology of mature *P. polyphylla* is depicted in Fig. 1, which emphasizes these key characteristics.

### Taxonomic system

**Figure 2:**
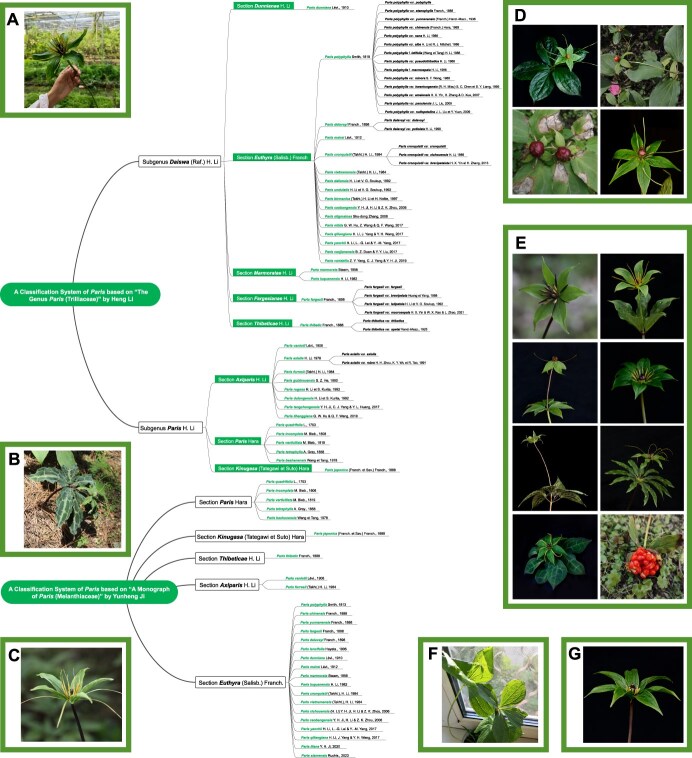
Two representative taxonomic systems for the genus *Paris.* The upper part presents a classification system of *Paris* based on ‘The Genus *Paris* (Trilliaceae)’ by Heng Li, where she categorizes *Paris* into two subgenera and eight sections based on various characteristics, including ovary placentation types. This system encompasses 34 species, 21 varieties and forms. The lower part shows the classification system based on ‘A Monograph of *Paris* (Melanthiaceae)’ by Yunheng Ji, who classifies *Paris* into five sections using ribosomal and chloroplast phylogenetics. This system accounts for 27 species. **A** Section *Dunnianae*, which is incorporated into Li’s system. **B** Section *Marmoratae*, which is incorporated into Li’s system. **C** Section *Thibeticae*, which is incorporated into Li’s and Ji’s system. **D** Section *Axiparis*, which is incorporated into Li’s and Ji’s system. **E** Section *Euthyra*, which is incorporated into Li’s and Ji’s system. **F** Section *Paris*, which is incorporated into Li’s and Ji’s system. **G** Section *Fargesianae*, which is incorporated into Li’s system.

The genus *Paris* Linn. was first established by the Swedish botanist Carolus Linnaeus in 1753 in *Species Plantarum*, with *P. quadrifolia* designated as the type species [17]. In 1789, De-Jusseau classified *Paris* within Liliaceae [18], a view subsequently adopted by several taxonomists [2]. In 2006, Wendy B. Zomlefer and colleagues reassigned *Paris* to Melanthiaceae based on molecular and morphological data [19], a classification reflected in the subsequent APG (Angiosperm Phylogeny Group) system. Taxonomic systems for *Paris* have been proposed by Franchet, Hara, Takhtajan, Li, and Ji, each presenting different schemes [2]. Chinese botanist Li classified the genus into two subgenera, *Daiswa* and *Paris*, based on ovary placentation types. Further, she divided *Paris* into eight sections—*Dunnianae*, *Euthyra*, *Marmoratae*, *Fargesianae*, *Thibeticae*, *Axiparis*, and *Paris*—based on characteristics such as stamen number, aril presence, and fruit dehiscence, encompassing 24 species, 14 varieties and forms [15]. Ji, another Chinese botanist, classified *Paris* into five sections based on ribosomal and chloroplast phylogenetics: *Paris*, *Kinugasa*, *Thibeticae*, *Axiparis*, and *Euthyra*, covering 26 species [2]. The taxonomic systems from both monographs, along with new species and varieties described in recent publications, are illustrated in Fig. 2, and the references are provided in Supplementary Data Note S1. Li’s system includes 34 species, 21 varieties and forms, while Ji's encompasses 27 species. With advancements in molecular biology, more scholars are exploring the systematic taxonomy of *Paris* at the molecular level. Nevertheless, the taxonomic system of the genus remains contentious, with potential resolutions anticipated with future scientific advancements.

### Geographic distribution and population status

**Figure 3:**
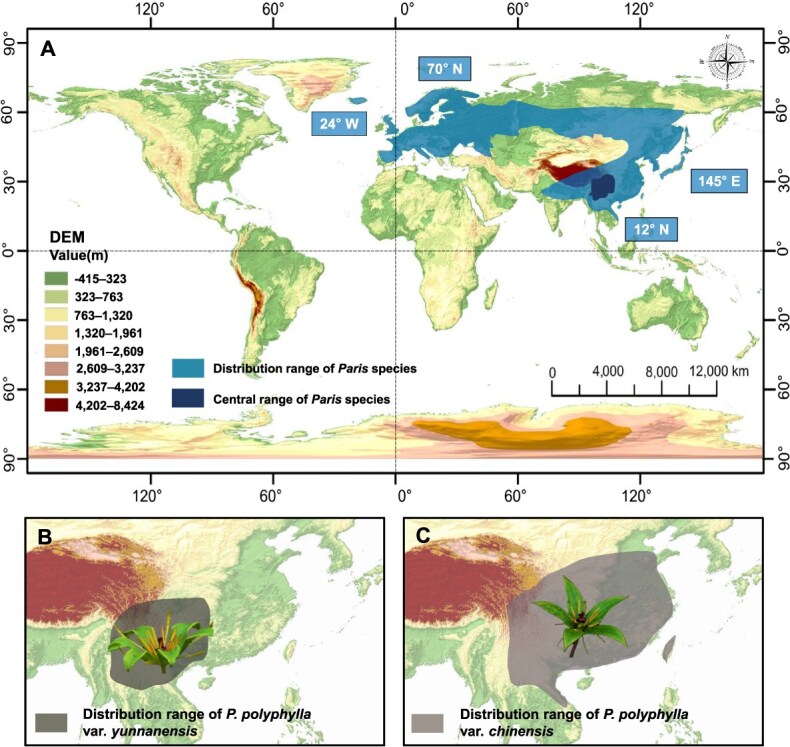
Geographic distribution of *Paris* species. **A** The cyan blue area represents the global geographic distribution range of *Paris* species, primarily located on the Eurasian continent, extending northwards to the Arctic (~70° N) and southwards to Indochina (~12° N), westwards to Iceland (~24° W), and eastwards to the Okhotsk region of Russia and Japan (~145° E). The dark blue area denotes the central range of *Paris* species, mainly encompassing Yunnan, Sichuan, Guizhou, and Chongqing provinces in southwest China. DEM represents the digital elevation model and the color ranges indicate altitude from −415 to 8424 m. **B** The dark taupe area indicates the global distribution range of *P. polyphylla* var. *yunnanensis*, predominantly in southwest China and neighboring countries, including Myanmar, Thailand, Laos, and Vietnam. **C** The medium taupe area shows the global distribution range of *P. polyphylla* var. *chinensis*, primarily in central and southern China, including Taiwan, as well as neighboring countries such as Myanmar, Thailand, Laos, and Vietnam. The world map is derived from WorldClim, https://www.worldclim.org/, with data sourced from the Shuttle Radar Topography Mission elevation data, and some elements designed by Evasplace/Freepik.

*Paris* species are primarily distributed across the Eurasian continent, ranging northwards to the Arctic (~70° N) and southwards to Indochina (~12° N), westwards to Iceland (~24° W), and eastwards to the Okhotsk region of Russia and Japan (~145° E) [2, 15, 20–22] (Global Biodiversity Information Facility, GBIF, https://www.gbif.org/, last access date 8 October 2024; Botanical Society of Britain and Ireland, BSBI, https://bsbi.org/, last access date 8 October 2024). *Paris* species are distributed across various altitudes, ranging from 0 to 4300 m, the majority occurring in low- to mid-altitude regions below 2000 m worldwide. The center of diversity for *Paris* species is located in the Yunnan, Sichuan, Guizhou, and Chongqing provinces of southwest China, spanning the mid- to high-altitude regions from the Yunnan–Guizhou Plateau to the Qionglai Mountains in Sichuan. The representative species *P. polyphylla* var. *yunnanensis* is distributed in southwest China and neighboring countries, including Myanmar, Thailand, Laos, and Vietnam, while *P. polyphylla* var. *chinensis* is distributed in central and southern China, including Taiwan, as well as neighboring countries, such as Myanmar, Thailand, Laos, and Vietnam [2, 15, 20, 22]. Figure 3 illustrates the distribution range and central region of *Paris* species, along with the geographic distribution of the representative species. *Paris* species thrive in a variety of habitats, including evergreen broadleaf forests, deciduous broadleaf forests, coniferous forests, bamboo groves, shrublands, grasslands, and riverbanks. These plants prefer growing in humus-rich soils or fertile sandy loam.

The phrase 'Chonglou, with its golden threads, is found everywhere, thriving in the damp and shaded mountains' (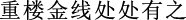, 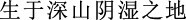) from *Compendium of Herbology* (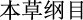, AD 1552–1578) suggests that the resources of *Paris* species were once abundant in China. However, currently, most wild populations of *Paris* exist in small, isolated groups, with limited numbers. Larger populations are typically found in remote areas or designated conservation zones, with human activity being the primary factor affecting their size. Since 2013, the soaring prices of RP in China have spurred massive wild harvesting of *Paris* populations by farmers [23]. The excessive and indiscriminate harvesting has severely damaged the diversity of wild resources of *Paris* populations and has also affected other plants with similar morphologies (such as *Trillium*) [2, 24]. This phenomenon has also spread to neighboring countries, such as Thailand, Nepal, Vietnam, and India [22]. Regarding the population trends of wild *Paris*, there is a dearth of quantitative data available, although some anecdotal and qualitative publications indicate population declines [6].

In response to the decline in population of wild *Paris*, China has established a series of conservation regulations, with 44 species and varieties of *Paris* listed as national second-class protected plants (https://www.plantplus.cn/rep/protlist, last access date 9 October 2024). Among these species, seven are classified as Vulnerable (VU), eight as Endangered (EN), and four as Critically Endangered (CR), including *P. dulongensis*, *P. luquanensis*, *P. undulatis*, and *P. wenxianensis*. According to the IUCN Red List website (https://www.iucnredlist.org/, last accessed 9 October 2024), *P. forrestii* is categorized as Least Concern (LC), and *P. polyphylla* as VU.

### Pollination and reproduction

Current studies indicate that *Paris* species exhibit a mixed mating system, incorporating both self-pollination and cross-pollination mechanisms, facilitated by both wind and insect vectors [2, 15, 25]. However, the efficiency of pollen transfer to stigmas is compromised due to excessive pollen wastage [24]. The flowers of *Paris* species lack nectaries and are typically unscented and non-vibrant in coloration (with exceptions like *P. japonica* and some populations of *P. delavayi*), rendering them less attractive to conventional pollinators such as bees and butterflies. Some researchers have noted their particular attractiveness to fly-like insects [24, 26], and there have been observations suggesting that spiders may play a role in aiding self-pollination [25].

Species of *Paris* are perennial, with rhizomes subsisting underground and serving as the primary nutrient supply organ for the entire plant. The rhizomes possess a terminal bud (some have several adventitious buds) and adventitious roots. These buds grow into new plants during favorable periods (usually in spring), while the aerial parts wither after flowering and fruiting (typically in autumn and winter). By this time, a new terminal bud has already formed, protected by a bud sheath. In a few species, such as *P. vanioti* and *P. fargesii* var. *latipelata*, the aerial parts do not die in winter but wither only after the emergence of new stems in the following spring. After the aerial part dies, a distinct scar is left on the rhizome, which can be used to approximate the age of *Paris* species [6]*.*

**Figure 4:**
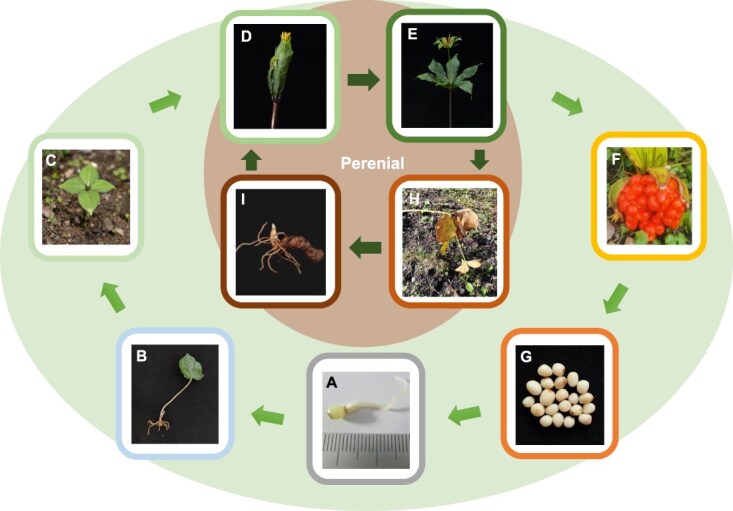
Illustration of a representative reproductive cycle of *P. polyphylla. Paris polyphylla* is a perennial plant. The brown circle illustrates the process of asexual reproduction in mature plants while the green circle represents the sexual reproduction spanning ~4*–*8 years. **A** Germinated seeds of *P. polyphylla*. **B** In the first year post-germination, *P. polyphylla* typically develops a single heart-shaped leaf. **C** In the second year after sprouting, *P. polyphylla* typically possesses four leaves. **D** Beginning in the fourth year, the plant enters the reproductive growth phase, the seedling of *P. polyphylla* being enveloped by leaves surrounding the flower prior to blooming. **E** Mature plant of *P. polyphylla* during the pollination period. **F** The mature fruit of *P. polyphylla* splits open, revealing seeds covered with a red aril. **G** White seeds of *P. polyphylla* with red aril removed. **H** The aerial part of *P. polyphylla* dies every winter. **I** After the aerial part of *P. polyphylla* dies, its apical bud will continue to develop and grow in the following year.

In natural settings, most *Paris* species demonstrate low seed germination rates and have a prolonged growth period, typically requiring at least 4–5 years to progress from seed to bloom [2, 15]. The incomplete embryo development and a certain dormancy period in their seeds necessitate experiencing two winters before sprouting. To shorten the germination time, warm–cold stratification treatment is commonly employed in cultivation, effectively reducing the period by several months. In the first year post-germination, *P. polyphylla* typically develops a single heart-shaped leaf, progressing to three or four leaves in the second year, and more than four in the third year [15]. The plant generally enters its reproductive phase in the fourth year, with a few individuals flowering while the majority flower and fruit between the fifth and seventh year, completing a full growth cycle. Figure 4 shows the representative reproductive cycle of *P. polyphylla*.

The high demand for *Paris* species, particularly for medicinal use, has driven advancements in propagation methods and techniques. Currently, two primary original plants of RP listed in the Chinese Pharmacopoeia, *P. polyphylla* var. *yunnanensis* and *P. polyphylla* var. *chinensis*, have well-established breeding and cultivation techniques. Traditional propagation still relies on rhizome cutting to maintain the genetic uniformity and quality of RP, despite this method resulting in low propagation rates and reduced net rhizome yields [27–29]. Recent studies have reported advances in developing *in vitro* micropropagation techniques of *Paris* species. Callus suspension cultures hold potential to replace the destructive harvesting of wild *Paris* resources while rapidly producing active phytochemicals such as steroidal saponins [28]. Moreover, somatic embryogenesis offers a promising micropropagation system that can produce genetically uniform *Paris* plantlets without undesirable traits. These plantlets effectively bypass the dormancy period and reduce production time by ~13 months [27]. Overall, the slow natural reproduction of *Paris* species remains a major challenge for the conservation and sustainable utilization of its resources. Future research should focus on leveraging biotechnology to enhance propagation methods and ensure a stable supply of high-quality RP.

## Medicinal value of *Paris* species

### Traditional medical perspective

In traditional medical systems like TCM, Indian Ayurvedic medicine, and Greco-Roman medicine, *Paris* species have been documented for their therapeutic uses. In TCM, *Paris* species, referred to as ‘Zao Xiu’ (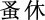), were first mentioned in *Shennong's Classic of Materia Medica* during the Eastern Han dynasty. The term 'Zao' refers to parasites, such as fleas, while 'Xiu' means 'to stop or rest', emphasizing the primary efficacy of *Paris* species in expelling parasites and treating venomous insect bites. Table 1 summarizes the representative traditional medicinal records concerning *Paris* species [26, 30, 31]. The historical texts consistently highlight the use of *Paris* species in treating snake and insect bites and infections.

**Table 1:** Representative traditional medicinal records concerning *Paris* species.

Ancient medical system	Classical text	Recorded name	Origin	Primary effects
Traditional Chinese medicine	Shennong's Classic of Materia Medica ( 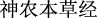 , AD 200)	Zao Xiu	*Paris* spp.	Expelling parasites and treating venomous insect bites, providing therapeutic benefits for epilepsy, spasms, digestive system inflammations, and skin infections
	Southern Yunnan Materia Medica ( 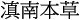 , AD 1436)	Chong Lou	*P. polyphylla* var. *yunnanensis* or closely related species	Against various swellings, infections, inflammations and alleviating difficulties in urination
	Compendium of Herbology ( 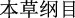 , AD 1552–1578)	Zao Xiu	*P. polyphylla* var. *polyphylla* and *P. polyphylla* var. *stenophylla*	Treating skin and soft tissue infections and throat inflammations
Indian Ayurvedic	Charaka Samhita ( 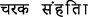 , B.C. 210 to AD 176)	Parseek vacha	*P. polyphylla*	Treating digestive issues, arthritis, neuralgia, anxiety, and insomnia
	Bhav Prakash ( 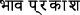 , AD 16th century)	Swet vachh		
Greco-Roman medicine	Commentary on the Six Books of Pedanius Dioscorides on Materia Medica (Commentarii in sex libros Pedacii Dioscoridis de Materia medica, A.D. 1554)	Herba Paris	*P. quadrifolia*	Not documented

### Modern medical perspective

*Paris* species are widely utilized in modern clinical practices, particularly in the treatment of inflammation conditions and skin diseases. For example, *Chonglou senbo* lotion combined with *Fuyanling* suppository has been effective in treating acute vaginitis. Similarly, *Zini chonglou* decoction paired with *Xiaokui* powder has been shown to reduce serum levels of TNF-α and IL-6, thereby alleviating symptoms in patients with ulcerative colitis. Furthermore, *Chonglou jiedu* decoction, when used in conjunction with retention enemas, can significantly relieve anal pain in patients with early-stage perianal abscesses and exhibits a low recurrence rate. For skin-related diseases, *Chonglou jiedu* tincture has demonstrated efficacy in treating neonatal toxic erythema, being both potent and easy to apply, and it can also be used in conjunction with tretinoin cream to treat flat warts. RP combined with Herba Euphorbiae Humifusae and Rhizoma Dioscoreae Nipponicae has been proved to be effective for herpes zoster. Additionally, RP combined with *Nidus Vespae* can significantly improve symptoms in patients experiencing postoperative gastric cancer recurrence. Post-cesarean administration of *Chonglou shenghua* decoction has been shown to significantly reduce the occurrence of persistent lochia while demonstrating a good safety profile (references are provided in Supplementary Data Note S2).

RP has been used in over 106 classic formulations for treating a wide variety of conditions (https://db.yaozh.com, last accessed 4 October 2024). These formulations are administered either topically or orally. For instance, *Gongxuening* capsules are used for treating heavy menstrual bleeding, postpartum hemorrhage, and other gynecological disorders. *Yunnan Baiyao* aerosol is used for bruises, contusions, muscle soreness, and other related symptoms. *Gufengning* capsules are indicated for the treatment of rheumatoid arthritis and ankylosing spondylitis. *Jidesheng sheyao* tablets are effective against venomous snake and insect bites. *Yinbing xiaocuo* tincture is employed for acne treatment. *Chonglou jiedu* tincture is used to treat herpes, skin itch, dermatitis caused by insect bites, and mumps. *Sanjie zhitong* cream helps alleviate cystic hyperplasia of the mammary glands and breast pain. *Fufang yanlian* tablets are used to address the common cold, cough, acute and chronic bronchitis, pharyngitis, and tonsillitis. *Houshu kouhan* tablets serve as a medicine against sore throat, throat itchiness, and dry throat. *Yunnan hongyao* powder is used to treat gastric ulcer bleeding, blood-stained sputum caused by bronchiectasis, bleeding hemorrhoids, soft tissue contusions, and rheumatoid arthritis. Additionally, some skincare brands incorporate RP and its extracts into their product lines to leverage its anti-acne property.

### Ethnobotanical applications

*Paris* species are widely used in folk medicine across the globe, especially in China and the Indochina region. Ethnobotanical applications of *Paris* species often focus on the rhizome, which is the most commonly used part in traditional medicine. Across various cultures, *Paris* rhizome is primarily used for treating a range of ailments, including inflammation, skin diseases, and gastrointestinal disorders. Additionally, it is recognized for its effectiveness in treating snake and insect bites, and promoting wound healing. Table 2 presents representative ethnobotanical applications of *Paris* species across different regions with references provided in Supplementary Data Note S3. Widespread usage in folk medicine demonstrates the cultural and historical importance of *Paris* species and provides a foundation for further research into their pharmacological potential and therapeutic applications.

**Table 2:** Representative ethnobotanical applications of *Paris* species globally.

Country and province		Species used	Part used	Primary effects
China	Yunnan	*Paris* spp.	Rhizome	Treating rheumatism, furuncles, sore throat, carbuncles, swelling, fractures, gastroenteritis, poisonous insect bites, neurological headaches, tonsillitis, and breast hyperplasia, among others
	Sichuan	*P. polyphylla*, *P. polyphylla* var. *stenophylla*, *P. polyphylla* var. *yunnanensis*, and *P. mairei*	Rhizome and flower	Treating cuts, stomach ailments, and edema
	Guizhou	*P. polyphylla* var. *yunnanensis*, *P. vaniotii*, and *P. daliensis*	Rhizome	Treating mumps, dysentery, otitis media, and duodenal ulcers
	Hainan	*P. dunniana*	Rhizome	Treating snakebite
	Inner Mongolia	*P. verticillata*	Rhizome	Treating furuncles, snakebites, lung-heat cough, mastitis, appendicitis, and infantile convulsions, among others
	Tibet	*P. polyphylla*	Rhizome	Treating diabetes
India		*P. polyphylla*	Rhizome, stem, and leaf	Preventing diarrhea and stomach pain, bandaging fresh cuts
Nepal		*P. polyphylla*	Rhizome	Treating fever, food poisoning, and snake and insect bites, as well as diarrhea and dysentery in cattle
Vietnam		*Paris* spp.	Rhizome	Treating fever, malaria, snakebites, acne, mastitis, tuberculosis, and asthma
Thailand		*P. polyphylla* var. *chinensis*	Rhizome	Reducing fever, healing wounds, and relaxing muscles
Korea		*P. verticillata*	Rhizome	Treating asthma, furuncles, and chronic bronchitis
UK		*P. quadrifolia*	Rhizome and fruit	The rhizome is used to treat wounds and induce vomiting and as an antidote for arsenic and mercury poisoning, while its fruit is used to treat eye inflammation

## Secondary metabolites, pharmacological activities, and toxicity evaluation of *Paris* species

### Secondary metabolites

Medicinally active substances in plants are often secondary metabolites [32]. Since 1960, over 330 secondary metabolites have been isolated and identified from different tissues of *Paris* species, such as rhizomes, roots, aerial stems, and leaves (references are provided in Supplementary Data Note S4). Steroidal saponins are the main bioactive components in *Paris* plants [33–39], accounting for more than 80% of the total identified compounds, which can be further divided into spirostanol, isospirostanol, furostanol, and pseudospirostanol according to the configuration of C-25 spirosteranes and the cyclic state of the F ring [8]. The representative steroidal saponins include polyphyllins I, II, V, VI, VII, and H, dioscin, gracillin, and trillin. In particular, polyphyllins I, II, and VII are considered as standards for evaluating the quality of RP in the Chinese Pharmacopoeia of Commission 2020, with a required content of no less than 0.60% [3]. These steroidal saponins with high polarity and relatively poor water solubility are primarily extracted from the rhizomes and leaves of *P. polyphylla* var. *yunnanensis* and *P. polyphylla* var. *chinensis* using methods like ethanol reflux extraction and ultrasonic-assisted extraction [40, 41]. More sustainable techniques, such as microwave-assisted extraction [42], water-assisted extraction [43], and deep eutectic solvents combined with ultrasonic-assisted extraction [44, 45], are also being explored. Traditional methods like thin-layer chromatography, ultraviolet–visible spectrophotometry, infrared spectroscopy, high-performance liquid chromatography, and gas chromatography are commonly used for the analysis of steroidal saponins and other compounds in *Paris* species. Over the past decade or so, modern analytical techniques, such as liquid chromatography–mass spectrometry, especially ultra-performance liquid chromatography coupled with quadrupole time-of-flight mass spectrometry (UPLC-Q-TOF-MS/MS), have been utilized to analyze secondary metabolites in *Paris* species [35, 46–50]. These techniques offer higher sensitivity, specificity, and accuracy, allowing more precise identification and quantification of bioactive compounds.

In addition to steroidal saponins, various other secondary metabolites have been isolated from *Paris* species, such as steroids [34, 37, 50–55], terpenoids [34, 51, 56], flavonoids [57, 58], and other types [59, 60]. Steroids identified include daucosterol, β-sitosterol and stigmasterol. Terpenoids include lupeol, paritrisides A–F and 3β-ol-oleane-12-en-28-oic acid-3-*O*-Glc(1 → 2)-Ara. Flavonoids include kaempferol, quercetin, and rutin. Other types include polysaccharides, verticillatins A–C, gallic acid, and falcarindiol.

### Pharmacological activities

**Figure 5:**
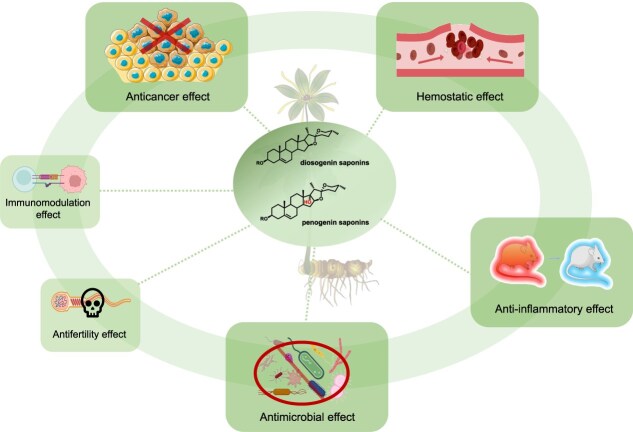
Pharmacological activities of steroidal saponins from *Paris* species. Contemporary pharmacological research demonstrates that steroidal saponins from *Paris* species typically possess anticancer, hemostatic, anti-inflammation, antimicrobial, antifertility, and immunomodulation effects. The materials in the figure are partly sourced from BioRender.com.

As the most significant active substances, the steroidal saponins of *Paris* species exhibit various pharmacological activities, such as anticancer, hemostatic, anti-inflammatory, antimicrobial, and other effects [10, 48, 53, 61–66]. The pharmacological activities of steroidal saponins from *Paris* species are illustrated in Fig. 5, and detailed information is presented in Supplementary Data Table S1. Numerous studies have highlighted the significant anticancer activity of steroidal saponins from *Paris* species, showing potential in treating various types of cancer, such as breast cancer, oral cancer, lung cancer, gastric cancer, colorectal cancer, and liver cancer. For example, polyphyllin II has been reported to eliminate cancer stem-like cell populations through inhibition of the Ras-related nuclear protein [67]. Similarly, polyphyllin VII has been shown to induce apoptosis in oral cancer cells by activating ERK, Akt, p38 MAPK, and JNK [68]. In addition to their anticancer effect, *Paris* species are also known for their hemostatic properties, the primary hemostatic compounds being pennogenin saponins. These saponins induce platelet aggregation by activating glycoprotein α_IIb_β_3_, dependent on extracellular calcium, ADP secretion, and thromboxane synthesis [69].

Other secondary metabolites in *Paris* species have also been demonstrated to possess significant pharmacological activities. For instance, the total flavonoids extracted from the aerial stems and leaves of *P. polyphylla* var. *yunnanensis* exhibited DPPH scavenging activity with IC_50_ of 0.12 mg/mL [70]. Kaempferol extracted from *Paris* species can reduce tumor angiogenesis by inhibiting vascular endothelial growth factor expression and prevent tumor cell metastasis by suppressing matrix metalloproteinases [71]. Lupeol, a triterpenoid found in *Paris* species, has been reported to exert anti-inflammatory effects by targeting multiple molecular pathways, including NF-κB, cFLIP, Fas, Kras, PI3K/Akt, and Wnt/β-catenin [72]. Moreover, a homogenous polysaccharide obtained from *P. polyphylla* leaves showed significant anti-aging effects by reducing oxidative damage and enhancing antioxidant defenses in a d-galactose-induced aging mouse model [73].

In summary, secondary metabolites in *Paris* species, predominantly steroidal saponins, exhibit a diverse array of pharmacological properties including anticancer, hemostatic, anti-inflammatory, antimicrobial, and other effects, making *Paris* species highly valuable medicinal plants. Additionally, as described in the ‘Medicinal value of *Paris* species’ section, both traditional modern medicine and ethnobotanical applications highlight the efficacy of *Paris* species in treating conditions such as bleeding, inflammation, skin diseases, swelling, and wound healing. This aligns with the pharmacological effects of their secondary metabolites on hemostasis, anti-inflammation, and antimicrobial aspects. However, based on the available literature, the majority of modern studies have focused on the anticancer effect of steroidal saponins from *Paris* species, diverging from traditional usage and clinical practice. This bias may be due to the more developed cancer models or different efficacy between the entire medicinal materials and isolated chemical compounds. Future research is expected to bridge this gap and explore the broader pharmacological applications based on their traditional usage and clinical practice.

### Toxicity evaluation

RP is noted in the Chinese Pharmacopoeia for its mild toxicity [3], and modern toxicological studies have confirmed that *Paris* species exhibit hepatotoxic, hemolytic, and reproductive toxicities. Specifically, RP saponins exhibit liver toxicity in SD rats by inhibiting fatty acid oxidation, glycolysis, and the TCA cycle, which leads to hepatocyte damage and dysfunction in lipoprotein transport. The underlying mechanism involves the downregulation of mRNA levels for CYP1A2, CYP2E1, and UDP-dependent glycosyltransferase (UGTs) [74]. Extracts from the leaves of *P. luquanensis* have shown significant toxicity to zebrafish embryos, reducing survival rates and increasing malformations [75]. Total saponins from *P. forrestii* exhibited acute hepatotoxicity in mice, with the LD_50_ of 92.40 mg/kg, and caused hemolytic toxicity, with the ED_50_ of 4.31 μg/mL [76]. Polyphyllin I was identified to show relatively high toxicity, inducing hepatocellular toxicity in HepaRG and HL-7702 cells via apoptosis [77], as well as causing hemolysis and eryptosis in human red blood cells through mechanisms involving elevated cytosolic Ca^2+^ levels and membrane permeabilization [78].

Despite these toxicological findings, *Paris* species are generally regarded as having low acute toxicity, with relatively high LD_50_ values. To date, no severe adverse effects or fatalities have been reported due to the consumption of *Paris* species under typical therapeutic conditions, suggesting that their overall toxicity to humans is considered minimal when used appropriately.

## Advances in molecular biology research on *Paris* species

**Figure 6:**
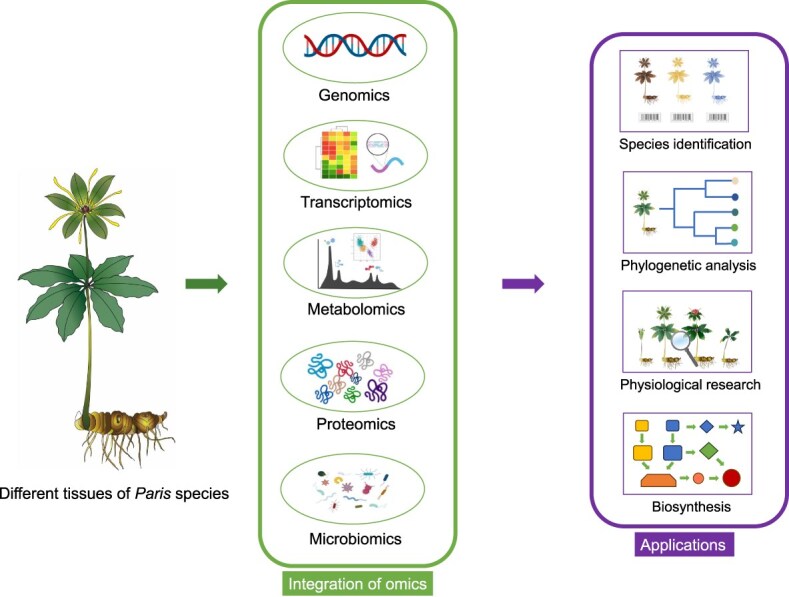
Integrated multi-omics approaches to addressing scientific issues related to the genus *Paris*. Recent progress in high-performance computing, particularly with the integration of multi-omics data, presents opportunities to surmount the challenges inherent in *Paris* species research. The confluence of diverse omics approaches, such as the combined analysis of genomes and transcriptomes, plays a pivotal role in the identification of species and the elucidation of their evolutionary trajectories. Furthermore, the application of metabolomics enables the characterization of metabolites in *Paris* species across different developmental stages and tissue types. This is instrumental in analyzing the patterns of accumulation and the underlying physiological mechanisms of polyphyllin, offering critical insights for its artificial cultivation. The incorporation of microbiomic data introduces a novel dimension to our understanding of the mechanisms underpinning the formation of secondary metabolites in *Paris* species from a phytochemical ecological standpoint. This perspective is valuable for grasping the biological significance of compounds exhibiting antimicrobial activity. Moreover, a comprehensive analysis of the biosynthetic pathways of polyphyllin is expected to facilitate its heterologous and efficient production, safeguarding the wild plant resources of *Paris* species. The materials in the figure are partly sourced from BioRender.com.

The genus *Paris*, characterized by its large genomes, presents a formidable yet intriguing challenge in the realm of molecular biology research. Recent advancements in genome sequencing technologies, coupled with the integrative use of genomics, transcriptomics, metabolomics, proteomics, and microbiomics, have led to considerable progress in elucidating the genetic diversity, systematic taxonomy, and functional genomics of *Paris* species. These representative studies furnish critical insights into species identification, phylogenetic relationships, plant physiological mechanisms, and the elucidation of biosynthetic pathways for pivotal secondary metabolites (Fig. 6).

### Application of molecular marker techniques in *Paris* species

Molecular marker techniques, including SSR, EST-SSR, ISSR, Scot, SRAP, and ALFP, have proven valuable for assessing the genetic diversity and population structure of *Paris* species, facilitating the accurate identification of these species and controlling the quality of RP [79–86]. DNA barcoding, employing markers such as psbA-trnH, trnL-trnF, and ITS, either individually or in combination, has become a widely adopted method for species identification within *Paris* [87–90]. Furthermore, ‘ultra-barcoding’ or genomic identification, has proven to be highly effective. Through comprehensive sequencing of whole plastid and ribosomal DNA, a cryptic species, *P. liiana*, was distinguished within *P. yunnanensis* [91]. Additionally, a comprehensive reference library comprising complete plastomes and high-resolution nrDNA arrays of *Paris* has also been established [92]. Building on this, a PCR-free genome skimming approach has been developed, facilitating the identification of *Paris* species at the seedling and rhizome stages [93].

### Genome sequencing efforts: unraveling large genomic mysteries

The genus *Paris* is notable within angiosperms for comprising species with significantly large genomes [94]. *Paris japonica*, an octoploid species within the genus, was reported to have the second largest known eukaryotic genome (148.89 Gbp/1C), smaller than the currently known largest eukaryotic genome of *Tmesipteris oblanceolata* (160.45 Gbp/1C) [94, 95]. Even *P. bashanensis*, the species with the smallest genome in the genus, boasts a genome size of 29.38 pg [2], significantly larger than the majority of angiosperms [96]. The underpinnings of these expansive genomes in *Paris* species are yet to be fully elucidated, with ancient polyploidy and chromosomal rearrangements posited as potential factors [2]. In 2020, a landmark achievement saw the assembly of 70.18 Gb of the 82.55-Gb genome of *P. polyphylla* var. *yunnanensis*, marking the largest genome sequenced to date [97]. This assembly highlighted that 69.53% of the *P. polyphylla* genome comprises repeat sequences, with long terminal repeat (LTR) transposable elements accounting for 62.50% of these repeats. The genomic characteristics of *Paris* species render it an exemplary model for studying genome size variation, despite the inherent challenges.

In parallel, the chloroplast genomes of *Paris* species, much simpler compared with their nuclear counterparts, have been extensively leveraged for species-level research. The complete chloroplast genome of *P. verticillata* was sequenced in 2014 and spans 157 379 bp [98]. To date, a total of 167 chloroplast genome sequences of *Paris* species have been published, exhibiting lengths ranging from 155 957 to 165 623 bp (https://www.ncbi.nlm.nih.gov/, last access date 9 October 2024). These chloroplast genomes are remarkably conserved in size, structure, gene content, and organization, facilitating phylogenetic reconstructions that shed light on the evolutionary relationships and divergence times among *Paris* species [2, 99–101]. Nonetheless, instances of cytonuclear discordance, indicative of ancient and recent hybridization events within the genus, complicate phylogenetic interpretations, underscoring the complexity of *Paris* genetics. The continuous decline in sequencing costs and advances in sequencing technology and assembly technology promise further exploration of the *Paris* genomes, offering profound implications for the conservation and utilization of *Paris* species and yielding novel insights into plant biology and evolution [91, 100].

### Investigating plant physiological mechanisms

Understanding the dormancy mechanisms of seeds and rhizomes represents a key area of focus in the physiological studies of *Paris* species. Transcriptomic analyses of seeds from *P. polyphylla* var. *yunnanensis* have identified genes associated with seed dormancy, including *UGT*s, *GA20ox*, *GA3ox*, *SAUR*, *ARF*, *CYP707A*, *NCED*, *ABI2*, *PP2C*, *ARP3*, *ARP7*, and *SUS*, among others [102–106]. Moreover, it has been demonstrated that gibberellic acid 3 (GA3) effectively breaks dormancy and significantly promotes multiple shoot formation in *P. polyphylla*, thereby boosting reproductive efficacy [29]. Proteomic studies suggest that the interplay between GA and abscisic acid is critical for *P. polyphylla* var. *yunnanensis* seed germination [107]. Additional research on *P. polyphylla* var. *chinensis* indicates that seed development and germination are encouraged by warm temperatures and hindered by cold conditions [108].

The application of multi-omics techniques is crucial for dissecting the complex processes of growth, development, and metabolism in *Paris* species. Utilizing SWATH-MS and GC/TOF-MS to assess the proteomes and metabolomes of rhizomes from three *Paris* species revealed that *P. polyphylla* var. *chinensis* exhibits superior efficiency in sucrose utilization within sugar metabolism pathways and boasts the highest protein abundance [109]. Investigations into the effects of red and blue light on leaf photosynthesis in *P. polyphylla* var. *yunnanensis* through metabolomics have demonstrated that a combination of red and blue light markedly enhances metabolite levels associated with carbon–nitrogen balance, energy metabolism, and secondary metabolism [110]. Rhizospheric and endophytic microorganisms play an essential role in regulating the accumulation of secondary metabolites in medicinal plants [111], and the dynamic interaction between *Paris* plants and rhizosphere soil microbes has also captured scientific interest. Studies on the microbial communities in the rhizomes and rhizosphere soil of *P. polyphylla* revealed that microbial diversity was shaped by environmental conditions and planting duration, potentially affecting the annual accumulation of active compounds in the plant [112–115]. Furthermore, Su *et al*. recently launched the inaugural global data portal for *Paris*, the PPDP, utilizing PacBio SMRT-based and Illumina-based RNA-Seq data. This portal serves as a valuable resource for *Paris* studies [116].

### Leveraging synthetic biology for *Paris* resources sustainable utilization

*Paris* species hold significant medicinal value but face severe challenges in resource conservation due to their lengthy growth cycle and excessive wild harvesting. Synthetic biology has emerged as an alternative approach for resource acquisition, enabling the rapid and eco-friendly production of large quantities of desired bioactive compounds. This approach shows great promise in the development and sustainable utilization of medicinal plant resources. Through the application of biotechnological techniques involving microorganisms or model plant chassis cells for enzymatic conversion, several natural products, including artemisinic acid [117], ginsenosides [118], and other natural products [119–121], have been successfully produced. However, applying this method to synthesize polyphyllins requires a comprehensive understanding of their biosynthetic pathways.

**Figure 7:**
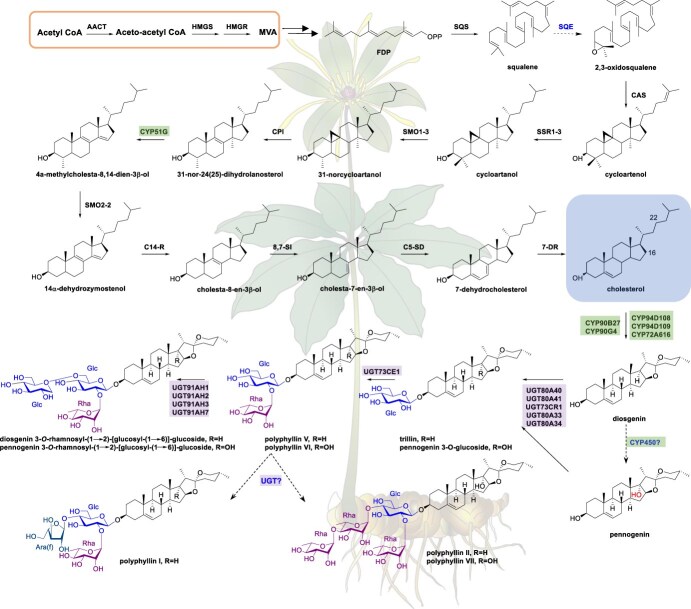
Proposed biosynthetic pathway of polyphyllins in *P. polyphylla*. AACT, acetyl CoA acetyltransferase; HMGS, 3-hydroxy-3-methylglutaryl-CoA synthase; HMGR, 3-hydroxy-3-methylglutaryl-CoA reductase; SQS, squalene synthase; SQE, squalene epoxidase; CAS, cycloartenol synthase; SSR1–3, sterol side chain reductase 1–3; SMO, C-4 sterol methyl oxidase; CPI, cyclopropylsterol isomerase; CYP51G, sterol C-14 demethylase; C14-R, sterol C-14 reductase; 8,7-SI, sterol 8,7 isomerase; C5-SD, sterol C-5(6) desaturase; 7-DR, 7-dehydrocholesterol reductase; CYP, cytochromes P450; UGT, UDP-glycosyltransferase. Enzymes in the steroid metabolic pathway with known functions are shown in black; enzymes with unknown functions are marked in blue. Black solid lines indicate established metabolic pathways; black dashed lines indicate speculated metabolic pathways.

It is widely accepted that cholesterol, synthesized via the cytoplasmic mevalonate (MVA) pathway, serves as a direct precursor for polyphyllins [122–125]. The cholesterol side chain undergoes a series of oxidative modifications to yield two primary aglycones, diosgenin and pennogenin (C17α-OH diosgenin), culminating in the formation of polyphyllins I, II, VI, and VII [123, 126–131]. Figure 7 illustrates the proposed biosynthetic pathway of polyphyllins in *P. polyphylla*.

Recent identification of several cytochrome P450 enzymes (CYPs) from *P. polyphylla* (PpCYP90G4, PpCYP94D108, PpCYP94D109, PpCYP72A616) has highlighted their critical roles in the polyphyllin biosynthetic pathway [123, 128]. PpCYP90B27 is a steroid C-22 hydroxylase, catalyzing the formation of 22*R*-hydroxycholesterol from cholesterol [128]. PpCYP90G4 is capable of catalyzing C-16 and C-22 dihydroxylation of cholesterol to generate 16*S*,22*S*-dihydroxycholesterol and subsequent oxidative ring closure bridging C16 and C22, while PpCYP94D108, PpCYP94D109 and PpCYP72A616 likely catalyze 27-hydroxylation triggering stereospecific formation of the terminal heterocycle to yield diosgenin. In addition, the P450 enzymes involved in the biosynthesis of diosgenin from cholesterol have been functionally characterized from *Trigonella foenum-graecum* and *Dioscorea zingiberensis*, two plants also producing diosgenin. Interestingly, 16*S*,22*R*-dihydroxycholesterol rather than 16*S*,22*S*-dihydroxycholesterol was demonstrated to be an intermediate of diosgenin biosynthesis [124]. Co-expression of these *P450* genes along with the rate-limiting enzymes of cholesterol biosynthesis in tobacco (*Nicotiana benthamiana*) or yeast has demonstrated the ability to synthesize diosgenin [122, 123, 132]. This research has led to the development of highly efficient systems for cholesterol synthesis, achieving yields of 5.63 mg/g dry weight and 10 mg/L in tobacco and yeast, respectively [122, 132].

UGTs are instrumental in generating complex and bioactive polyphyllins. A series of UGTs from *P. polyphylla* var. *yunnanensis* (UGT80A33, UGT80A34, UGT80A40, UGT80A41, UGT73CR1, UGT73CE1, UGT91AH1, UGT91AH2, UGT91AH3, and UGT91AH7) have been functionally characterized, playing pivotal roles in polyphyllin glycosylation [133–135]. Among them, UGT80A33, UGT80A34, UGT80A40, UGT80A41, and UGT73CR1 are sterol 3-*O*-*β*-glucosyltransferases, catalyzing the formation of the monoglycosides diosgenin 3-*O*-glucoside (trillin) and pennogenin 3-*O*-glucoside. UGT73CE1 is a steroid glucoside 2′-*O*-*β*-rhamnosyltransferase, catalyzing the glycosylation of monoglycosides to generate polyphyllins V and VI. Four steroid glucoside 6′-*O*-glucosyltrasferases, namely UGT91AH1, UGT91AH2, UGT91AH3, and UGT91AH3, catalyze the glycosylation of diglycosides to form diosgenin/pennogenin 3-*O*-rhamnosyl-(1 → 2)-[glucosyl-(1 → 6)]-glucosides, which could be detectable but have yet to be isolated from *Paris* species.

In addition, the biosynthesis of active compounds in medicinal plants is intricately regulated by various transcription factors (TFs), and insights into the regulatory mechanisms governing polyphyllin biosynthesis promise to identify potential targets for heterologous synthesis [136]. Transcriptome analyses across different tissues from *Paris* species and other plants have identified several TF-encoding genes implicated in polyphyllin synthesis, spanning gene families such as *bHLH*, *WRKY*, *MYB*, *AP2/ERF*, *bZIP*, and *NAC* [129, 137].

Due to the lacks of a high-quality reference genome and a suitable system for genetic manipulation, investigating the biosynthesis and regulation of active ingredients in *Paris* species remains challenging. Although a series of CYPs and UGTs have been characterized from *Paris* species, there are still some limitations due to the complexity of plant secondary metabolism and the multitiered regulation mechanisms. For instance, it is yet to be determined whether 16*S*,22*R*-dihydroxycholesterol or 16*S*,22*S*-dihydroxycholesterol is the real intermediate, and which UGTs are responsible for polyphyllin biosynthesis *in planta*. In addition, the enzymes responsible for the C-17 hydroxylation reaction remain unidentified. Further in-depth investigation into the biosynthesis and synthetic biology of polyphyllins is urgent; such work not only lays the groundwork for developing new pharmaceuticals but also provides innovative solutions to address the sustainable utilization challenges of medicinal *Paris* resources.

## Concluding remarks and future perspectives

The genus *Paris* is distinguished by its remarkable morphology and medicinal attributes, boasting both a lengthy history of medicinal utilization and status as a rare, distinctive botanical resource. A review of recent literature reveals that investigations into *Paris* species primarily focus on resource identification, compound characterization, and pharmacological activity analysis. The exploration of functional genes within the genus *Paris* is still in its infancy, in sharp contrast with the dynamic advancements in molecular genetics research in model plants. This raises several pertinent questions. Do the phylogenetic characteristics, pollination mechanisms, seed dormancy, and other botanical behaviors observed in *Paris* species align with those documented in model plants? Furthermore, given the expansive genomes of *Paris*, what factors govern its size and complexity? And, importantly, how many genetic resources within *Paris* species remain unexplored?

The study of polyphyllin biosynthesis has represented a significant focus within the genus *Paris* in recent years, with several crucial enzyme genes successfully cloned and functionally characterized. By leveraging insights from biosynthetic pathways, metabolic engineering facilitates the low-cost production of high-value active compounds through heterologous biosynthesis in microorganisms or plants. This sustainable production approach is expected to mitigate the excessive exploitation of wild plant resources. Nonetheless, considerable knowledge gaps persist in the comprehensive analysis and reconstitution of the polyphyllin biosynthetic pathway. Furthermore, the mechanisms underlying the biosynthesis, transport, and accumulation of these valuable active compounds within the plant remain unclear, including the external environmental factors and internal genes that govern these intricate and complex processes. Therefore, further studies are needed to enhance the understanding of the molecular mechanisms underlying the biosynthesis and transcriptional regulation of active compounds in the genus *Paris*.

The advent of next-generation high-throughput sequencing technologies has dramatically reduced sequencing costs, paving the way for the acquisition of high-quality reference genomes for *Paris* species. Concurrently, advances in gene transcription methodologies are being progressively applied to medicinal plants. A notable example includes the development of a high-resolution road map in *Catharanthus roseus* leaves through single-cell RNA sequencing [138, 139]. This innovative approach enables the detection of gene expression at the single-cell level, providing novel insights into the biosynthesis, transport, and storage mechanisms of active compounds. Furthermore, the integrative analysis of metabolomics and transcriptomics enhances our comprehension of plant metabolic regulatory networks. Beyond their application in model plants such as rice [140] and tomato [141], multi-omics strategies have also been employed to elucidate the spatial distribution and molecular mechanisms underlying the major active metabolites in the root and leaf tissues of medicinal plants like danshen (*Salvia miltiorrhiza*) [142]. It is anticipated that multi-omics analysis, spatial metabolomics, and mass spectrometry imaging (MSI) techniques will become increasingly prevalent in *Paris* research, heralding a new era of exploration into the complex molecular landscapes of these medicinal plants.

Gene editing technologies have marked a pivotal advancement in the field of model plant genetics research. However, traditional gene delivery methods have encountered limited success in medicinal plants. The recent implementation of the cut-dip-budding gene delivery system in medicinal plants like dihuang (*Rehmannia glutinosa*) and yuanzhi (*Polygala tenuifolia*), which were previously considered challenging or unfeasible targets for gene editing, represents a significant breakthrough [143]. This innovative approach, which obviates the need for sterile conditions and tissue culture, not only broadens the spectrum of plants amenable to straightforward transformation and genetic modification but also heralds the potential for its widespread application across various medicinal plants. Moreover, successful heterogeneous grafting using the medicinal plant *Artemisia annua* as the scion and the model plant tobacco as the rootstock has also provided new approaches for exploring metabolic regulatory genes in complex medicinal plants and for targeted breeding [144]. Consequently, the ongoing refinement of plant biotechnology techniques is poised to significantly bolster genetics research and molecular breeding efforts in *Paris* species and beyond.

## Supplementary Material

Web_Material_uhae327
