# Supplementary material for: The genus Paris: a fascinating resource for medicinal and botanical studies
Source: Hortic Res. 2024 Nov 21;12(3):uhae327. doi: 10.1093/hr/uhae327 (PMC11883231; doi:10.1093/hr/uhae327)
Supplement: Web_Material_uhae327 [file web_material_uhae327.zip › HR-2024-688_Supplementary materials.docx]

**Note S1**

Two major monographs on the genus *Paris* and all publications describing new species within this genus [1-16]. The information is summarized in Figure 2.

**Note S2**

Eight publications describing the modern clinical practices of *Paris* species [17-24], indicating that *Paris* species are mainly used to treat inflammation and skin diseases.

**Note S3**

11 publications describing the ethnobotanical applications of *Paris* species globally [25-35], in combination with our own field investigations, indicating that *Paris* rhizome is primarily used in folk medicine for treating a range of ailments, including inflammation, skin diseases, and gastrointestinal disorders. The information is summarized in Table 2

**Note S4**

25 publications describing the isolation and identification of specialized metabolites from the genus *Paris* [36-60]. The conclusions are presented in the main text.

**Table S1: Pharmacological activities of** **steroidal saponins of *Paris* species.**

| Pharmacological activitie | Compounds/  Extracts | Material/model | Mechanism | Results | Reference |
| --- | --- | --- | --- | --- | --- |
| Anticancer effect | Polyphyllin VII (also named polyphyllin G) | Circulating tumor cell line TJH-01 and lung cancer cell line H1975 | Downregulating TrkB and EGFR-MEK/ERK pathways | Inducing anoikis; inhibiting the migration | [61] |
|  |  | Immunodeficient mouse models |  | Reducing lung metastasis and circulating tumor cell numbers |  |
|  |  | Oral cancer cells SAS and OECM-1 | Activating ERK, Akt, p38 MAPK, and JNK pathways | Inducing apoptosis via caspase activation and PARP cleavage; promoting autophagy by increasing LC3-II and beclin-1 expression | [62] |
|  | Polyphyllin II (also named polyphyllin B) | Breast cancer cell lines MDA-MB-231, MDA-MB-436, and MCF-7 | Activating the Hippo signaling pathway by binding to the MST2-MOB1-LATS1 complex | Inducing caspase-dependent apoptosis; promoting autophagy by regulating YAP and LATS1 activity | [63] |
|  |  | MDA-MB-231 xenograft mouse model |  | Suppressing tumor growth; regulating pLATS1, LC3-II, Beclin 1, YAP, p62, and Ki67 levels in tumor tissue |  |
|  |  | Fibrosarcoma cell line HT-1080 | Activating PKM2, and inhibiting the expression of Glut1, LDHA and MYC | Inducing oxidative stress, mitochondrial dysfunction, and apoptosis | [64] |
|  |  | Gastric cancer cell lines | Down-regulating GPx4 and promoting NCOA4-dependent iron autophagy and Fe²⁺ accumulation via TFR1 | Inducing apoptosis and cell cycle arrest; promoting ferroptosis | [65] |
|  |  | Orthotopic mouse model |  | Suppressing tumor growth |  |
|  |  | Cancer stem-like cells (CSCs) enriched triple-negative breast cancer (TNBC) tumor cells | Binding directly to Ras-related nuclear protein, and inhibiting its function in GTPase-mediated pathways | Reducing stemness markers (Sox2, Nanog); inhibiting tumor sphere formation | [66] |
|  |  | CSCs enriched TNBC tumor mouse models |  | Inhibiting primary tumor growth, post-surgical recurrence and lung metastasis |  |
|  | Polyphyllin Ι (also named polyphyllin D) | Colorectal cancer cell line HCT-116 | Downregulating antioxidant proteins and regulating cell cycle proteins such as p21 and cyclinB1 | Decreasing cell viability; inducing ROS-dependent autophagy; causing G2/M phase arrest | [67] |
|  |  | Hepatocellular carcinoma cell lines | Modulating the Nrf2/HO-1/GPX4 axis | Inducing ferroptosis; enhancing mitochondrial disruption; | [68] |
|  |  | Nude mouse xenograft model |  | Inhibiting tumor growth |  |
| Hemostatic effect | Total steroidal saponins | Human and rat platelets | Activating α_IIb_β_3_ integrin, involving calcium influx, ADP secretion, thromboxane synthesis and PI3K pathway | Inducing dose-dependent platelet aggregation | [69] |
|  |  | Mice and rats |  | Promoting hemostasis |  |
|  | Polyphyllin H | Plasma from rats | Releasing ADP and producing TXA_2_ | Inducing platelet aggregation in a dose-dependent manner | [70] |
|  |  | Mice | Binding to thrombin | Increasing fibrinogen levels; shortening bleeding time |  |
|  | Pennogenin tetraglycoside | Rat platelets | Activating P2Y1 and P2Y12 receptor signaling pathways | Inducing platelet shape change, aggregation, and secretion of dense and α-granules | [71] |
| Anti-inflammatory effect | Polyphyllin H and polyphyllin H enriched extract | LPS-induced RAW264.7 and HaCaT cells | Binding to KEAP1, activating NRF2 and upregulating MAPK pathway | Suppressing IL-1β and IL-6 expression, ROS generation, NF-κB translocation; upregulating HO-1 | [72] |
|  | Polyphyllin VI | Mice with chronic neuroinflammatory pain | Downregulating P2X_3_ receptor expression | Decreasing paw withdrawal latency and threshold; reducing foot edema; lowering the expression of IL-1, IL-6 and TNF-α | [73] |
|  | Polyphyllin VII | LPS- induced RAW264.7 cells | Suppressing NF-κB and MAPK pathways | Reducing NO and PGE2 production; decreasing mRNA and protein expression of pro-inflammatory cytokines and enzymes | [74] |
|  |  | Mice and zebrafish embryos |  | Inhibiting ear edema and granuloma formation in mice; suppressing inflammation and toxicity in zebrafish embryos |  |
|  | Polyphyllin I | LPS/IFN-γ-induced bone marrow-derived macrophages and peritoneal elucidated macrophages | Inhibiting NF-κB pathway | Reducing pro-inflammatory cytokines; inhibiting IKKα/β and p65 phosphorylation; blocking p65 nuclear translocation | [75] |
|  |  | Collagen-induced arthritis mouse model |  | Attenuating bone erosion, synovitis, and infiltration of M1-like macrophages and T cells |  |
| Antimicrobial effect | Polyphyllin I, II, V, VI, VII, H, and gracillin | Fungal strains: *Trichophyton rubrum*, *Epidermophyton floccosum*, *Microsporum gypseum* and *Candida albicans* | Disrupting fungal cell membranes via an ergosterol-dependent mechanism | Inhibiting fungal growth, fungal spore germination; damaging fungal cell membranes | [76] |
|  | Polyphyllin VII | Bacterial strains such as *Proteus mirabilis* and *Staphylococcus aureus*  Fungal strains such as *Candida albicans* and *Trichophyton rubrum* | Not documented | Inhibiting bacterial and fungal growth | [77] |
|  | Ethanol extract | Enterovirus 71 and Coxsackievirus B3 strains | Not documented | Increasing IL-6 production; inhibiting viral infection; inactivating viruses | [78] |
| Antifertility effect | Pennogenin saponins and dioscins extracts | Mice | Not documented | Exhibiting spermicidal activity | [79] |
| Immunomodula-tion effect | Polyphyllin II | CD4^+^CD25^+^ T cells from lupus nephritis patients | Not documented | Increasing TGF-β and IL-10 levels | [80] |

**Supplementary References**

1. Ji YH, Yang CJ & Huang YL. A new species of *Paris* sect. Axiparis (Melanthiaceae) from Yunnan, China. *Phytotaxa*. 2017;**306**:234–6

2. Liu YY, Luo DD, Yao H*. et al.* A new species of *Paris* (Melanthiaceae) from Yunnan, China. *Phytotaxa*. 2017;**326**:297–300

3. Ruchisansakun S, Sraphet S, Yothawut C*. et al.* Revision on the Genus *Paris* in Thailand, with a New Species *Paris* siamensis. *Plants (Basel)*. 2023;**12**:430

4. Zhang SD, Wang H & Li DZ. A New Species of *Paris* (Melanthiaceae) from Northeastern Yunnan, China. *Novon: A Journal for Botanical Nomenclature*. 2008;**18**:550-4

5. Wang Z, Cai XZ, Zhong ZX*. et al.* *Paris nitida* (Melanthiaceae), a new species from Hubei and Hunan, China. *Phytotaxa*. 2017;**314**:145-9

6. Xu Z, Wei N, Tan Y*. et al.* *Paris lihengiana* (Melanthiaceae: Parideae), a new species from Yunnan, China. *Phytotaxa*. 2019;**392**:045-53

7. Yang ZY, Yang CJ & Ji YH. *Paris variabilis* (Melanthiaceae), a new species from southwestern China. *Phytotaxa*. 2019;**401**:190-8

8. Yin HX, Zhang H & Xue D. *Paris polyphylla* var*. emeiensis* H. X. Yin, H. Zhang D. Xue, a new variety of Trilliaceae from Sichuan, China. *Acta Phytotaxonomica Sinica*. 2007;**45**:822–7 (in Chinese)

9. Yuan Y & Liu JL. A new variety from Sichuan, China—*Paris polyphylla* var. *nullopetalina* (Trilliaceae). *Applied Mathematics and Nonlinear Sciences*. 2022;**7**:957-64

10. Chen SC & Liang SY. A New Combination of *Paris*. *Journal of Systematics and Evolution*. 1995;**33**:490 (in Chinese)

11. He SZ. A new medicinal species of *Paris* from Guizhou. *Guizhou Science*. 1990;**8**:16-8 (in Chinese)

12. Liu JL. *Paris polyphylla* var.*panxiensis* (Trilliaceae), A New Variety from Sichuan, China. *Acta Botanica Boreali-Occidentalia Sinica*. 2009;**29**:169-1700 (in Chinese)

13. Wang SF. A new variety Of *Paris* from Sichuan. *Bulletin of Botanical Research*. 1988;**8**:139-42 (in Chinese)

14. Yin HX & Zhang H. *Paris cronquistii* (Takht.) H.Li var. *brevipetalata* H.X.Yin et H. Zhang, A New Variety of *Paris* (Trilliaceae）from Sichuan, China. *Acta Botanica Boreali-Occidentalia Sinica*. 2013;**33**:190-3 (in Chinese)

15. Rao WX, Xue D, Zhang KY*. et al.* Biological characteristics supplement and systematic position of *Paris cronquistii* var. *xichouensis Guihaia* 2018;**38**:602－7 (in Chinese)

16. Zhao L, Wang QH, Ren ZX*. et al.* *Paris fargesii* Franch. var. *macrosepala*, A New Variety of *Paris* L. (Melanthiaceae) from Hunan, China. *Journal of Tropical and Subtropical Botany*. 2022;**30**:543-8 (in Chinese)

17. Yuan QY. Clinical observation on the treatment of acute vaginitis using a combination of Chonglou, Kusen, Huangbai wash and Fuyanling Suppository. *China's Naturopathy*. 2022;**30**:94-6 (in Chinese)

18. Mao LH. Clinical observation on Chonglou decoction combined with Xiaokui powder in treating ulcerative colitis of dampness-heat accumulated in interior type. *Chinese Medicine Modern Distance Education oF China*. 2021;**19**:102-4 (in Chinese)

19. Chen Q, Zhou Y, Zhang M*. et al.* Clinical study on modified Chonglou jiedu decoction on early perianal abscess *Journal of Emergency in Traditional Chinese Medicine*. 2020;**29**:1168-71 (in Chinese)

20. Yang L. Clinical observation of 50 cases of neonatal toxic erythema treated by Chonglou Jiedu tincture coating. *Journal of Pediatrics of Traditional Chinese Medicine*. 2020;**16**:73-5 (in Chinese)

21. Cai XZ & Wang MH. Clinical efficacy observation of treating flat warts with topical application of Chonglou jiedu tincture combined with vitamin A acid cream. *Infection,Inflammation,Repair*. 2022;**23**:164-5 (in Chinese)

22. Geng WW, Qiao LP & Wang J. Professor Tong Xiaolin's experience in the treatment of herpes zoster. *Jilin Journal of Chinese Medicine*. 2022;**42**:1160-2 (in Chinese)

23. Zhang WJ. Brief discussion on the experience of treating malignant tumor with honeycomb and Chonglou. *Clinical Medicine*. 2019;133 (in Chinese)

24. Jiang YJ, Huang YP & Ye HJ. Clinical study on prevention of lochia after cesarean section by Chonglou shenghua decoction. *Chinese Journal of Clinical Pharmacology and Therapeutics*. 2014;**19**:437-41 (in Chinese)

25. LI HT, Guan YH, Ma J*. et al.* Folk usage of Chonglou in Xishuangbanna area of Yunnan province. *Journal of Medicine & Pharmacy of Chinese Minorities*. 2010;**2**:34 (in Chinese)

26. Qi L & Luo SD (eds.). Chinese minority traditional medicine Department. Inner Mongolia Science And Technology Press. Chifeng. 2000

27. Addi YW, Ren ZX, Rutherford S*. et al.* Ethnobotanical study on medicinal plants used by the Yi people in Xiaoliangshan, Yunnan Province, SW China. *J Ethnopharmacol*. 2024;**323**:117683

28. Ding YG, Zhao YL, Zhang J*. et al.* The traditional uses, phytochemistry, and pharmacological properties of *Paris* L. (Liliaceae): A review. *J Ethnopharmacol*. 2021;**278**:114293

29. Li S, Zhang Y, Guo YJ*. et al.* Monpa, memory, and change: an ethnobotanical study of plant use in Medog County, South-east Tibet, China. *J Ethnobiol Ethnomed*. 2020;**16**:5

30. Lalsangluaii F, Chinlampianga M & Shukla AC. Efficacy and potency of *Paris polyphylla* Smith, an ethno-medicinal plant of Mizoram. *Sci Technol J*. 2013;**1**:36-40

31. K.C.1 M, Phoboo S & Jha aPK. Ecological study of *Paris polyphylla* Sm. Ecoprint. *ECOPRINT*. 2010;

32. Nga NQ, Huyen PT, Truong PV*. et al.* Taxonomy of the genus *Paris* L. (Melanthiaceae) in Vietnam. *Tap Chi Sinh Hoc*. 2016;**38**:333-9

33. Ruamrungsri N, Siengdee P, Sringarm K*. et al.* *In vitro* cytotoxic screening of 31 crude extracts of Thai herbs on a chondrosarcoma cell line and primary chondrocytes and apoptotic effects of selected extracts. *In Vitro Cell Dev Biol Anim*. 2016;**52**:434-44

34. Jacquemyn H, Brys R & Hutchings MJ. Biological Flora of the British Isles: *Paris quadrifolia* L. *J Ecol*. 2008;**96**:833-44

35. Allen D & Hatfield G. Medicinal Plants in Folk Tradition− an Ethnobotany of Britain and Ireland. Timber Press. Oregon. 2004

36. Yan H, Ni W, Yu LL*. et al.* Parisvaniosides A-E, five new steroidal saponins from *Paris vaniotii*. *Steroids*. 2022;**177**:108949

37. Liu F, Li LN, Tian XC*. et al.* Chemical Constituents and Pharmacological Activities of Steroid Saponins Isolated from *Rhizoma Paridis*. *J Chem*. 2021;**2021**:1-7

38. Guo K, Ren X, Mu R-F*. et al.* Ecdysteroids and spirosterane steroids from the traditional Chinese medicine *Paris polyphylla* var. *yunnanensis*. *Phytochem Lett*. 2021;**45**:117-20

39. Guan LJ, Ding LS, Li YM*. et al.* A new homo-aro-cholestane glycoside from the rhizome of *Paris polyphylla* var. *chinensis*. *J Asian Nat Prod Res*. 2021;**23**:1107-14

40. Liu Y, Qiu PC, Wang MC*. et al.* New Steroidal Saponins Isolated from the Rhizomes of *Paris mairei*. *Molecules*. 2021;**26**:6366

41. Thapa CB, Paudel MR, Bhattarai HD*. et al.* Bioactive secondary metabolites in Paris polyphylla Sm. and their biological activities: A review. *Heliyon*. 2022;**8**:e08982

42. Yu LL, Wang S, Wang J*. et al.* Steroidal saponin components and their cancer cell cytotoxicity from *Paris rugosa*. *Phytochemistry*. 2022;**204**:113452

43. Yu LL, Li YX, Gao WT*. et al.* Steroidal saponins with cytotoxic activity from the stems and leaves of *Paris fargesii*. *New J Chem*. 2022;**46**:19136-46

44. Jiang XL, He Yj, Hou X, Y.*. et al.* Two new polyhydroxylated steroidal glycosides from *Paris polyphylla v*ar. *yunnanensis*. *Phytochem Lett*. 2022;**49**:171-6

45. Hu JM, Tang HF, Lu YY*. et al.* A new cyclocholestanol-type steroidal saponin from *Paris polyphylla* var. *stenophylla*. *Central South Pharmacy*. 2022;**20**:736-43 (in Chinese)

46. Hu JM, Lu YY, Zheng SX*. et al.* Steroid and triterpenoid saponins from the rhizomes of *Paris polyphylla* var. *stenophylla*. *Chin J Nat Med*. 2023;**21**:789-800

47. Liu Y, Liu MY, Bi LL*. et al.* Cytotoxic steroidal glycosides from the rhizomes of *Paris polyphylla* var. *yunnanensis*. *Phytochemistry*. 2023;**207**:113577

48. Liu Y, Tang N, Zhang Q*. et al.* Cytotoxic steroidal glycosides from the roots of *Paris verticillata* M.Bieb. *Phytochem Lett*. 2023;**53**:231-8

49. Li TY, Du Y, Wang MC*. et al.* Cytotoxic Steroidal Saponins Containing a Rare Fructosyl from the Rhizomes of *Paris polyphylla* var. *latifolia*. *Int J Mol Sci*. 2023;**24**:7149

50. Tian YY, Liu Y, Qiu PC*. et al.* Cytotoxic steroidal saponins from the rhizomes of *Paris fargesii* var. *Petiolata*. *Bioorg Chem*. 2023;**131**:106305

51. Liu Y, Tang N, Qian XY*. et al.* New steroidal saponins from the roots of *Paris verticillata*. *Nat Prod Res*. 2023;1-9

52. Li XH, Jiang W, Guo Y*. et al.* Steroidal sapogenins from the aerial parts of *Paris polyphylla* var. *yunnanensis* and activity evaluation. *Fitoterapia*. 2023;**167**:105498

53. Rawat JM, Pandey S, Rawat B*. et al.* Traditional Uses, Active Ingredients, and Biological Activities of *Paris polyphylla* Smith: A Comprehensive Review of an Important Himalayan Medicinal Plant. *J Chem*. 2023;**2023**:1-18

54. Dong X, Jiang W, Wan J-F*. et al.* Chemical constituents from stems and leaves of *Paris polyphylla* var. *yunnanensis*. *Chinese Traditional and Herbal Drugs*. 2023;**54**:2705-9 (in Chinese)

55. Zheng ZL, Tan XM, Guan LJ*. et al.* New steroidal saponins from aerial parts of *Paris polyphylla* var. *chinensis*. *China Journal of Chinese Materia Medica*. 2023;**48**:4589-97 (in Chinese)

56. Su HM, Nie W, Zhao B*. et al.* A New C_21_ Steroid Saponin from the Pericarps of *Paris polyphylla* var. *yunnanensis*. *Journal of Chinese Medicinal Materials*. 2024;**47**:85-90 (in Chinese)

57. Duan XY, Yue MC, Yang J*. et al.* Chemical constituents from *Paris rugosa* rhizomes and their antimicrobial activities. *China Journal of Chinese Materia Medica*. 2024;**48**:2981-8 (in Chinese)

58. Nie W, Zhang HY, Ma YX*. et al.* New steroidal saponins from the aerial parts of *Paris polyphylla* var. *yunnanensis* and their effects on blood coagulation. *Fitoterapia*. 2024;**174**:105833

59. Guan LJ, Zheng ZL, Guo ZY*. et al.* Steroidal saponins from rhizome of *Paris polyphylla* var. *chinensis* and their anti-inflammatory, cytotoxic effects. *Phytochemistry*. 2024;**219**:113994

60. Liu Y, Bi Y, Bai JT*. et al.* Steroidal saponins from the roots of *Paris verticillata* and their anti-proliferative and anti-inflammatory activities. *Phytochemistry*. 2024;**218**:113941

61. Que Z, Luo B, Yu P*. et al.* Polyphyllin VII induces CTC anoikis to inhibit lung cancer metastasis through EGFR pathway regulation. *Int J Biol Sci*. 2023;**19**:5204-17

62. Hsieh MJ, Chien SY, Lin JT*. et al.* Polyphyllin G induces apoptosis and autophagy cell death in human oral cancer cells. *Phytomedicine*. 2016;**23**:1545-54

63. Xiang YC, Peng P, Liu XW*. et al.* Paris saponin VII, a Hippo pathway activator, induces autophagy and exhibits therapeutic potential against human breast cancer cells. *Acta Pharmacol Sin*. 2022;**43**:1568-80

64. Wu J, Ding Z, Zhong M*. et al.* Polyphyllin II Induces Apoptosis in Fibrosarcoma Cells via Activating Pyruvate Kinase M2. *Chem Res Toxicol*. 2024;**37**:1394-403

65. Hu C, Zu D, Xu J*. et al.* Polyphyllin B Suppresses Gastric Tumor Growth by Modulating Iron Metabolism and Inducing Ferroptosis. *Int J Biol Sci*. 2023;**19**:1063-79

66. Wang K, Zhu S, Zhang Y*. et al.* Targeting the GTPase RAN by liposome delivery for tackling cancer stemness-emanated therapeutic resistance. *J Control Release*. 2024;**375**:589-600

67. Yu S, Wang L, Cao Z*. et al.* Anticancer effect of Polyphyllin Iota in colorectal cancer cells through ROS-dependent autophagy and G2/M arrest mechanisms. *Nat Prod Res*. 2018;**32**:1489-92

68. Yang R, Gao W, Wang Z*. et al.* Polyphyllin I induced ferroptosis to suppress the progression of hepatocellular carcinoma through activation of the mitochondrial dysfunction via Nrf2/HO-1/GPX4 axis. *Phytomedicine*. 2024;**122**:155135

69. Fu YL, Yu ZY, Tang XM*. et al.* Pennogenin glycosides with a spirostanol structure are strong platelet agonists: structural requirement for activity and mode of platelet agonist synergism. *J Thromb Haemost*. 2008;**6**:524-33

70. Cong Y, liu XL, Yu ZL*. et al.* Study on the effect and mechanism of Chonglouoside H on platelet aggregation. *Medical Journal of Chinese People's Liberation Army*. 2010;**35**:1429-32 (in Chinese)

71. Cong Y, Liu XL, Kang LP*. et al.* Pennogenin tetraglycoside stimulates secretion-dependent activation of rat platelets: evidence for critical roles of adenosine diphosphate receptor signal pathways. *Thromb Res*. 2012;**129**:e209-16

72. Yang Y, Wang CF, Wang J*. et al.* Rhizoma Paridis saponins attenuate Gram-negative bacteria-induced inflammatory acne by binding to KEAP1 and modulating Nrf2 and MAPK pathways. *J Cell Mol Med*. 2024;**28**:e18146

73. Luo ZH, Wang TT, Zhang ZL*. et al.* Polyphyllin VI screened from Chonglou by cell membrane immobilized chromatography relieves inflammatory pain by inhibiting inflammation and normalizing the expression of P2X(3) purinoceptor. *Front Pharmacol*. 2023;**14**:1117762

74. Zhang C, Li CY, Jia XJ*. et al.* *In Vitro* and I*n Vivo* Anti-Inflammatory Effects of Polyphyllin VII through Downregulating MAPK and NF-κB Pathways. *Molecules*. 2019;**24**:875

75. Wang Q, Zhou X, Zhao Y*. et al.* Polyphyllin I Ameliorates Collagen-Induced Arthritis by Suppressing the Inflammation Response in Macrophages Through the NF-kappaB Pathway. *Front Immunol*. 2018;**9**:2091

76. Chen YG, Yan Q, Ji YH*. et al.* Unraveling the serial glycosylation in the biosynthesis of steroidal saponins in the medicinal plant *Paris polyphylla* and their antifungal action. *Acta Pharmaceutica Sinica B*. 2023;**13**:4638-54

77. Cai X, Guo L, Pei F*. et al.* Polyphyllin G exhibits antimicrobial activity and exerts anticancer effects on human oral cancer OECM-1 cells by triggering G2/M cell cycle arrest by inactivating cdc25C-cdc2. *Arch Biochem Biophys*. 2018;**644**:93-9

78. Wang YC, Yi TY & Lin KH. *In vitro* activity of *Paris polyphylla* smith against enterovirus 71 and coxsackievirus B3 and its immune modulation. *Am J Chin Med*. 2011;**39**:1219-34

79. Shen F, Yang LJ & Peng YF. Study on the antifertility effect of the saponins in Rhizoma Paridis Yunnanensis *in vitro*. *Chinese Journal of Modern Applied Pharmacy*. 2010;**27**:961-4 (in Chinese)

80. Wang J, Liu RH & Xiao HB. The effects of Paridis saponin II on the production of cell cytokines in CD4^+^CD25^+^ T regulation cells from lupus nephritis patients' peripheral blood. *Progress in Modern Biomedicine* 2010;**10**:50-3 (in Chinese)
